# A Systematic Review of Healthcare-Associated Infectious Organisms in Medical Radiation Science Departments

**DOI:** 10.3390/healthcare8020080

**Published:** 2020-03-30

**Authors:** D’arcy Picton-Barnes, Manikam Pillay, David Lyall

**Affiliations:** 1 School of Health Sciences, The University of Newcastle, Callaghan 2308, Australia; d.picton-barnes@uon.edu.au (D.P.-B.); manikam.pillay@newcastle.edu.au (M.P.); 2 Centre for Resources Health and Safety, School of Health Sciences, The University of Newcastle, Callaghan 2308, Australia

**Keywords:** Healthcare-associated infection, occupational-associated infection, infectious organisms, medical radiation science, diagnostic radiography, radiation therapy, nuclear medicine

## Abstract

Healthcare-associated infections (HAIs) pose a significant occupational risk to medical radiation science (MRS) professionals, who have a high degree of patient contact. Current research largely focusses on HAIs in patients, with limited attention given to infectious organisms that MRS professionals are exposed to. This is a significant gap that this systematic review seeks to address by summarizing current literature to determine the infectious organisms within MRS departments, their reservoirs, and transmission modes. Reporting of this systematic review follows the preferred reporting items for systematic reviews and meta-analyses guidelines. Five databases were searched (Scopus, Medical Literature Analysis and Retrieval System Online (MEDLINE), the Cochrane Library, EMBASE, and Cumulative Index to Nursing and Allied Health Literature (CINAHL)) for relevant studies published between 1983 and 2018. Quality assessment was performed using checklists from the Johanna Briggs Institute. Nineteen studies were included in the review; twelve of which were set in diagnostic radiography departments, two in radiotherapy departments, and five in non-MRS departments. No studies were set in nuclear medicine departments, indicating a gap in the available literature. A total of 19 genera of infectious organisms were identified in the literature, with *Staphylococcus, Escherichia, Bacillus*, and *Corynebacterium* reported in all MRS departments. Infectious organisms were identified in all observational studies, indicating a need for better infection control methods and/or compliance training within MRS to minimize the risk of infections.

## 1. Introduction

Healthcare-associated infections (HAIs) arise from the colonization of microorganisms on a new host within a healthcare setting [1]. These include infectious organisms that patients are exposed to when receiving care, and carry occupational risks of infection among healthcare workers [1]. HAIs are problematic as they result in greater rates of mortality and morbidity, increase antimicrobial resistance in microorganisms, and also increase costs for healthcare systems and those receiving care [1]. Over the last century, developed countries have relied upon immunizations and antimicrobial medications as the primary methods of disease prevention and management [2]. However, with the rise of multi-drug resistant bacteria, the efficacy of these infection control methods has diminished [2]. More recently, focus has shifted to hygiene and sanitation practices as the primary solution for preventing the spread of these infections; aiming to reduce the occurrence of infections rather than treating infections post-colonization [1,3]. Despite improvements in these practices, HAIs are still prevalent in healthcare facilities, affecting hundreds of millions of patients worldwide each year [1]. Appropriate infection control guidelines, as well as healthcare worker compliance in applying these measures, are imperative in reducing the number of HAIs, thereby reducing their burden and improving both patient and staff outcomes [1]. Though many microbes (including viruses and fungi) are responsible for HAIs, where an estimated 90% are caused by bacteria, including species of *Staphylococcus*, *Escherichia*, *Enterococci*, and *Klebsiella* [4]. 

Different classes of bacteria behave differently in the presence of antimicrobial agents [5], therefore distinguishing between them is necessary to ensure appropriate infection control methods are being implemented, thus minimizing the occurrence of infections [5]. Using the Gram stain test, bacteria can be separated phenotypically into Gram-positive and Gram-negative [5]. The primary difference between Gram-positive and Gram-negative bacteria is the size and structure of the cell wall [5]. Gram-positive bacteria have a relatively thick, continuous peptidoglycan wall (20–80 nm) containing teichoic and lipoteichoic acids [5]. Gram-negative bacteria have a thin layer of peptidoglycan; an outer membrane that contains phospholipids; lipopolysaccharide; proteins; and while not directly involved in staining, Gram-negative bacteria have a third layer (inner membrane) [5]. These two layers are separated by a periplasmic space containing transport, degradative and cell-wall synthetic proteins [5]. The Gram-stain test involves staining bacteria with a crystal violet, attempting to remove the stain, and then staining any decolourized cells with a red stain (safranin) [5]. Because of the differing characteristics of the cell wall, Gram-positive bacteria trap the stain within the cross-linked structure of the peptidoglycan layer, turning them purple [5]. Conversely, the crystal violet is not retained by the thin peptidoglycan layer of Gram-negative bacteria, which washes off [5]. Accordingly, the presence of the outer membrane gives Gram-negative bacteria a barrier that prevents substances accessing the peptidoglycan layer, including antibacterial drugs and antimicrobial cleaning solutions [5]. Without this extra layer of protection, mild detergents have a greater effect on Gram-positive bacteria than Gram negative bacteria [5].

Medical radiation science (MRS) professionals have a high degree of direct contact with members of the community who are susceptible to infections including children, the elderly, and those with compromised immune systems [6]. This level of interaction means that if an MRS worker develops an infection, there is a high chance of it spreading to these susceptible patients (and similarly from patients to healthcare workers) [6]. In addition, a lot of the equipment utilized within MRS is shared between patients with a broad range of pathologies [6]. As such, appropriate hygiene practices are imperative to ensure this equipment does not harbor harmful microorganisms [6]. Reducing the risk of exposure to infectious organisms is the responsibility of both the healthcare institution and individual practitioners [1,7]. Most healthcare departments follow general infection control guidelines. For instance, the New South Wales Health Infection Prevention and Control Policy [8] specifies that shared patient equipment must be cleaned “according to manufacturer’s instructions” [8]. However, due to the high-risk nature of patients within MRS, more specific methods of infection control may be necessary in order to reduce the risk of HAIs. Furthermore, as different organisms require different methods of infection prevention and control [5], such as sterilization or disinfection, infection control methods should be specific to the nature of practice performed within the healthcare setting [1,5]. Research into the specific infectious organisms found within MRS departments may serve to guide infection control strategies.

Current research predominantly focuses on HAIs among patients only, and not on the occupational-associated infections (OAIs) that staff are exposed to. When addressing this gap, Nienhaus et al. reported that the annual rate of recognized OAIs in non-government hospitals in Germany were 15.3 per 100,000 healthcare workers [9]. This study did note that the data may have been incomplete as it relied upon healthcare workers reporting infections, and considerable underreporting of OAIs was likely to have occurred [9]. Nienhaus et al. concluded that the risk of OAIs remains high, and as such “awareness for the infection risk and knowledge about infection prevention should be improved” [9]. Along with limited research regarding OAIs in healthcare workers, the prevalence of infectious organisms across MRS professions has also been underexplored in the literature. As such, this study aims to address these research gaps and contribute to the available literature by determining the OAIs that MRS staff are most likely at risk of contracting within their departments. Having an understanding of infectious organisms in MRS departments is an important first step in establishing programs and approaches for reducing infection risks among healthcare workers, and consequently among patients [10]. This systematic review therefore aims to collect and analyze data from recent literature to determine the infectious organisms that MRS staff are exposed to within their departments; and examine the reservoirs and modes of transmission of these organisms. This review extends the scope of existent literature by including MRS, which has not been covered in previous research [10].

## 2. Materials and Methods 

A systematic review (SR) of the literature was performed to identify studies and establish which infectious organisms found in MRS have been identified in the literature, as the first part of an on-going research project on improving healthcare risk management in MRS departments. The methodology of this review was adapted from a previous SR analyzing the relationship between hospital staffing and HAIs [10]. Reporting of this SR follows the preferred reporting items for systematic reviews and meta-analyses (PRISMA) guidelines [11].

### 2.1. Protocol and Registration

The protocol for this SR was developed and registered to the international prospective register of systematic reviews (PROSPERO; registration number: CRD42019127575).

### 2.2. Search Strategy 

Electronic databases Scopus, EMBASE, the Cochrane Library, Medical Literature Analysis and Retrieval System Online (MEDLINE), and the Cumulative Index to Nursing and Allied Health Literature (CINAHL) were searched for relevant studies published between 1983 and 2018. The earliest date restriction for studies included in this SR was chosen as a number of international infection control documents were published in 1983, including the Association for Professionals in Infection Control and Epidemiology Curriculum [12]. The search was performed on 27 January 2019 and restricted to studies published in the English language. The search strategy (Table 1) was developed using a combination of medical subject headings (MeSH) and keywords associated with the topic, including “healthcare associated infection”, “hospital pathogens”, and “allied health.” Additional studies were identified through a manual search of citations in included studies. 

### 2.3. Selection Criteria 

All studies examining infectious organisms found directly in MRS departments, or outlining a risk of infection to MRS staff, were included. Studies on clothing or equipment commonly found in these departments were also included. Studies set in intensive care, critical care, surgical wards, or maternity wards were excluded. Case studies, non-peer reviewed literature, reviews, editorials, commentaries, articles on community-acquired infections, articles published prior to 1983, and articles not published in English language were also excluded. 

### 2.4. Definitions 

For the purpose of this review, the following definitions were used:

- Medical radiation scientists were defined as healthcare professionals that perform diagnostic imaging studies or plan and deliver radiation treatments to patients. Australian titles for these professionals include diagnostic radiographers, nuclear medicine scientists, and radiation therapists.
- Healthcare-associated infections (HAIs) comprise of infections acquired by persons in a healthcare setting [4]. These include bloodstream infections and organism-specific infections (such as methicillin-resistant *Staphylococcus aureus* and *Clostridium difficile*) that were defined as being associated with healthcare in the literature [4].
- Occupational-associated infections (OAIs) refer to infections that healthcare professionals acquire or are at risk of contracting from occupational exposure to infectious organisms within their departments.

### 2.5. Study Selection 

The titles and abstracts of all studies were assessed for relevance according to the SR aim, and articles that were not relevant were excluded. The full texts of remaining articles were acquired to further assess relevance based on the SR inclusion and exclusion and criteria. Articles with relevant study design and data were included. The study selection process was performed by researches using Covidence, an online SR management program. Two researches reviewed the title and abstracts of studies, checking the level of agreement at several intervals. The average level of inter-reviewer agreement was 93%. A third reviewer cross-checked included studies against the criteria at each stage of the study selection process. Any disagreements or discrepancies in the application of the selection criteria were resolved by discussion between all reviewers. 

### 2.6. Data Extraction 

Data was extracted into a Microsoft Excel spreadsheet. Data extracted from eligible studies included: title, author(s), year of publication, country, study population, healthcare department (setting), sample, type(s) of organisms, organism incidence and/or prevalence data, outcomes, and limitations. Extracted data were reviewed by a second researcher.

### 2.7. Risk of Bias 

Due to the varying study designs of included articles, checklists from The Joanna Briggs Institute (JBI) were utilized to assess content validity [13]. The results of each eligible study were recorded; however, these results were not used as a basis for exclusion. This assessment was performed independently by two reviewers, and the level of correlation was determined. 

### 2.8. Data Analysis 

Data from included studies were synthesized and displayed in evidence tables. Summary tables included studies that examined the prevalence of infectious organisms, specific department and infectious organisms, and equipment and infectious organisms. Reporting of organism classifications varied across the literature, and as such the data had to be analyzed further when formulating these tables. 

## 3. Results

### 3.1. Overview

The volume of literature related to infectious organisms within MRS departments was initially unknown, therefore the research question originally included all allied health professions as well as MRS. The search across five electronic databases returned 2063 studies, with an additional 8 studies identified through manual searching of reference lists. This number was reduced to 1676 once duplicates were excluded. A total of 1634 abstracts that did not focus on medical radiation science departments (or associated equipment) or focused on community-acquired infections were excluded at this stage.

**Figure 1:**
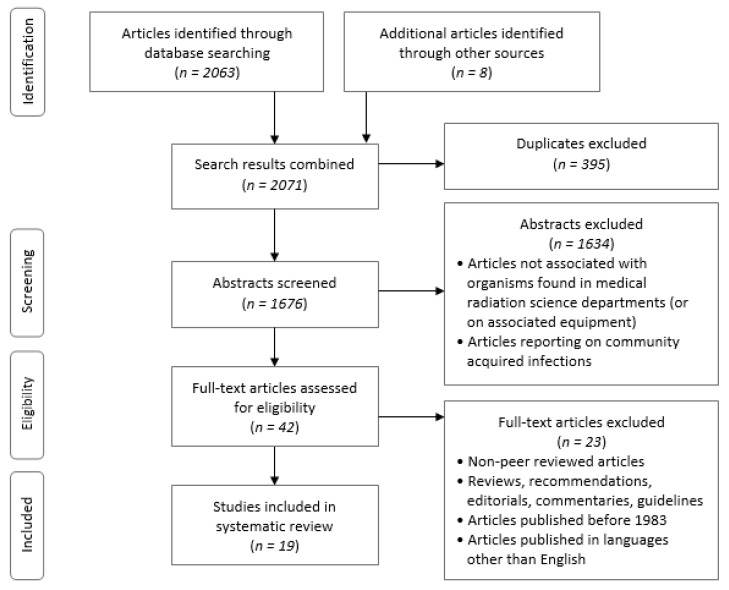
Preferred reporting items for systematic reviews and meta-analyses (PRISMA) flow diagram [11] depicting the stages of article identification, screening, assessment for eligibility, and final inclusion of 19 articles.

The full texts of the remaining 42 articles were acquired and screened against the inclusion and exclusion criteria, resulting in 19 articles eligible for review and evaluation [6,14,15,16,17,18,19,20,21,22,23,24,25,26,27,28,29,30,31]. This iterative process was carried out in accordance with the PRISMA statement [11] and is presented in Figure 1.

### 3.2. Characteristics of Included Studies

The final set of 19 included articles were published from Africa, Asia, North America, and Europe, and were published between 1998 and 2018. A total of 12 studies were set in diagnostic radiography departments, and two were set in radiotherapy departments. Five studies were of a non-specific or non-MRS setting but included information on equipment found in MRS (such as mobile phones, scissors, or identification badges). There were no studies set in nuclear medicine departments. 

Of the included studies, 15 were of observational design and aimed to identify the healthcare-associated organisms within MRS departments, or on equipment utilized in MRS [6,14,15,16,17,18,20,21,22,23,24,26,28,30,31]. An additional observational study was included as it identified the risk of occupational blood exposure, and therefore risk of infection to particular groups of healthcare workers [19]. The other three included studies were quasi-experimental, and were designed to test the ability of organisms to attach to and survive on equipment within MRS [25,27,29]. Table 2 describes the setting and healthcare equipment analyzed within the included studies, as well as the number of infectious organisms identified.

### 3.3. Infectious Organisms

Of the included studies, 15 examined the prevalence of specific organisms within MRS or on equipment found in MRS departments. There were 88 counts of infectious organisms reported across the included literature, which were then classified by their genus. A total of 19 different genera were identified (Table 3), as well as three counts of coliforms (a group of Gram-negative bacteria that includes the genera *Escherichia*, *Klebsiella*, *Enterobacter*, *Serratia*, and *Citrobacter*) and one count of unspecified diplococci (Gram-positive or Gram-negative bacteria that occur as pairs of cocci). Seven different genera of Gram-positive bacteria were identified, including *Staphylococcus*, *Micrococcus* and *Bacillus*; 10 different genera of Gram-negative bacteria were identified including *Acinetobacter* and *Escherichia*; as well as two genera of fungi (*Candida* and *Cladosporium*). 

At least one species of *Staphylococcus* was identified in every included study assessing contamination of healthcare departments, with the exception of one study [6] that expressed their results in terms of colony-forming units (CFUs) rather than specific organism(s). *Staphylococcus, Escherichia, Bacillus*, and *Corynebacterium* species, as well as coliforms other than *Escherichia*, were reported in all MRS departments studied, including diagnostic radiography, ultrasonography, and radiation therapy. The three most frequently identified infectious organisms were *Staphylococcus* (18.2%)*, Corynebacterium* (11.7%), and *Bacillus* (10.4%), all of which are Gram-positive. In total, 13 different genera of infectious organisms were reported in diagnostic radiography departments (plus one count of diplococci), 13 were reported in ultrasonography departments, and six were reported in radiation therapy departments. There were 16 different genera reported from other departments with *Vibrio* being the only organism not also identified in MRS departments (found on identification badges). With the exception of *Kocuria* (blood transmissible), all of the infectious organisms identified in the literature are transmissible via contact. Four of those organisms can also be transmitted through air.

### 3.4. Contamination of Equipment

A total of 31 different healthcare items were analyzed for infectious organisms within the included studies, with observational studies assessing 30 different items of equipment, and quasi-experimental studies assessing an additional item. Within this review, the majority of observational studies examining contamination of equipment were set in diagnostic radiography departments (*n* = 8). Consequently, most of the equipment analyzed (79.3%) was also located in diagnostic radiography departments, with radiographic cassettes (*n* = 3) and ultrasound probes (*n* = 3) being the most commonly evaluated sources of contamination. 

The studies included in this SR reported that a relatively large percentage of healthcare equipment used within MRS departments was contaminated with infectious microorganisms. Eight out of 15 studies (60%) noted that over 70% of sampled equipment was contaminated, with a pooled mean of 62.5% and a range of 13.6%–100%. Five studies reported the contamination to be less than 50%, with only one of those studies reporting contamination of less than 30%. Of all sampled equipment, identification badges were reported to have the lowest level of contamination (13.6%). The number of different infectious organisms identified on sampled equipment ranged from one to ten, with an average of five genera identified per study. 

Two of the included studies expressed the levels of contamination in terms of colony-forming units (CFUs). The number of CFUs present on sampled equipment ranged from none to more than 1000, with a pooled mean of 82.6 CFUs per sample. One article examining the levels of contamination on radiographic cassettes reported bacterial contamination on 95% of samples [20]. However, this study reported relatively low levels of contamination, with a range of 0–194 CFUs and a pooled mean of approximately 30 CFUs per cassette [20]. Another study examined the CFUs on a range of equipment utilized in diagnostic radiography departments, including x-ray tubes, control panels, imaging plates, and radiographic cassettes [6]. This study reported control panels to be the most heavily contaminated item, with a range of six to more than 1000 CFUs, and a pooled mean of 280.5 CFUs per control panel [6]. The pooled mean and ranges for each outcome measure evaluated are presented in Table 4, which was adapted from a study by Schabrun and Chipchase [32].

### 3.5. Methodological Quality

Quality assessment of studies was performed using JBI critical appraisal tools [13]. According to the hierarchy of evidence, all 19 studies included in this SR demonstrate the use of low (cross-sectional and quasi-experimental) to intermediate (cohort) quality research designs [33]. Of the 19 studies, 18 were of sound methodological quality, with the remaining article reported to have indeterminate quality. The appraisal results of each included study are reported in Table 5.

## 4. Discussion

This SR was aimed at investigating the different infectious organisms that MRS staff may be exposed to, their reservoirs, and their modes of transmission. The authors believe this is one of the first such reviews to focus on this specific context. The literature search resulted in the inclusion of 19 relevant studies. Of the 19 studies, 15 aimed to identify the healthcare-associated organisms found within MRS or on equipment utilized in MRS; one study investigated the risk of occupational blood exposure among diagnostic radiographers; and the other three assessed the potential for equipment used in MRS to act as a reservoir for infectious organisms.

### 4.1. Infectious Organisms

A total of 19 different genera were identified, as well as three counts of coliforms and one count of unspecified diplococci. These organisms included seven genera of Gram-positive bacteria, 10 genera of Gram-negative bacteria, and two genera of fungi. Out of the 88 organisms recorded in the literature, 64% (*n* = 56) were Gram-positive bacteria, 31% (*n* = 27) were Gram-negative bacteria, 5% (*n =* 4) were fungi, and 1% (*n =* 1) was unspecified bacteria. The majority of normal skin flora is comprised of Gram-positive bacteria (such as *Staphylococcus epidermidis* species), increasing the likelihood of it being transferred onto healthcare equipment during patient contact [28]. The inclusion of these environmental flora in some studies may explain the higher levels of Gram-positive bacteria observed on healthcare equipment. 

With respect to exposure of MRS staff to HAI organisms, many of those identified (such as normal flora) are considered “opportunistic”, whereby they generally will not cause infections in healthy humans. However, as Maczulak [34] pointed out, under certain conditions (including immunodeficiency, trauma, injury, or chronic disease), these opportunistic pathogens can cause infection. MRS professionals often utilize needles within their practice; for example, when administering contrast or radiopharmaceuticals, or when giving a patient positioning tattoos for radiotherapy. One of the included articles outlining the prevalence of needle-stick injures noted that 34 out of 814 (4.18%) of radiologists in the study group had experienced an occupational blood exposure at some point in their career [19]. Due to this inherent risk of blood exposure through needlestick injuries, many of these opportunistic infectious organisms (including *Staphylococcus, Kocuria*, and *Candida*) were considered to be OAIs and as such were included in results. Furthermore, with the steady increase of antibiotic-resistant bacteria, it can be expected that the prevalence of these serious infections among immunocompetent humans will continue to increase, further justifying their inclusion [34].

A range of both Gram-positive and Gram-negative bacteria were identified in all MRS departments, including *Staphylococcus, Bacillus, Escherichia*, and *Corynebacterium.* These results suggest that current infection control methods may not be effective in preventing the occurrence of significant pathogens. Of the 15 studies that identified the presence of *Staphylococcus*, 12 studies (80%) also reported the presence of *Staphylococcus aureus (S. aureus)*. *S. aureus* is considered a significant pathogen in healthcare settings, affecting both immunocompromised and immunocompetent individuals [4]. Methicillin-resistant *S. aureus* (MRSA) is a highly pathogenic strain of *S. aureus* that is resistant to antibiotics, significantly increasing the difficulties associated with controlling the spread of disease [4]. Fortunately, only one study [16] classified the identified species as MRSA. Of the three studies that did not report *S. aureus*, two studies reported counts of coagulase-negative *Staphylococcus*, and the other study reported *Staphylococcus* as a genus (failing to specify what species or strain was identified). As several of the studies failed to do further testing to determine antibiotic resistance, it is possible that the true prevalence of MRSA within MRS departments may have been underestimated. 

### 4.2. Healthcare Equipment as a Reservoir for Microorganisms

Previous research has identified healthcare equipment as a common source of infectious organism contamination [3,4]. A large proportion of sampled healthcare equipment was reported to be colonized with infectious organisms. The pooled mean of 62.5% contamination is concerning, indicating that over half of all healthcare equipment utilized in MRS harbors the infectious organisms responsible for OAIs. As previously mentioned in microbiology, CFUs approaching 100 may be considered heavy levels of contamination [20]. Giacometti et al. [6] reported a range of 6–1000 CFUs on equipment used in diagnostic radiography, with a pooled mean of 280.5 CFUs found on control panels. This result is concerning as it indicates that frequent points of contact for radiographers may be heavily contaminated, increasing the risk of OAIs. These findings were consistent across the majority of studies, indicating a need for more appropriate infection control approaches and/or compliance training. 

Conflicting results were observed from the included studies examining microbial contamination on mobile phones. [15,16] Bhat et al. [16] reported that 99% of all mobile phones assessed (201/204) were contaminated with bacteria, whereas Arora et al. [15] reported contamination in only 41% (65/160) of samples from mobile phones. The samples were acquired using similar swabbing techniques, culturing mediums and incubation periods, with the only obvious difference being the substances on the swabs used during data collection. Considering the variation in organism counts, the conclusion that mobile phones are a potential reservoir for infectious organisms is consistent. 

There were no counts of viral organisms recorded in the included literature, which could potentially be due to them not being tested for during data collection and analysis. Another explanation could be that viruses require a host to reproduce, whereas bacteria do not [35]. This greatly reduces the lifespan of viruses when compared to bacteria [35]. On equipment and surfaces, viruses have an average life expectancy of a few days, whereas bacteria can survive for months [35]. There are several practical reasons that may explain why viruses were not similarly examined for on MRS equipment. The largest being the relatively high cost of testing for viral contamination when compared to bacteria. In addition, there is a requirement for specialized medium, in vitro cell cultures/embryonated eggs, and low usage of high cost test kits (e.g., Enzyme Linked Immunosorbent Assay (ELISA)) with a short outdate, which means many hospital laboratories must send their viral cultures to a reference lab for analysis.

### 4.3. Transmission of Occupational-Associated Infections

The infectious organisms identified in the literature are transmissible through a number of routes, including air, contact, and blood. Out of the 19 genera of organisms identified, 18 are contact transmissible. This indicates that there is a high risk for these organisms to be transferred between patients, healthcare equipment, and MRS professionals. Four of the included studies evaluated the potential for organisms to attach and survive on equipment used in MRS. Hodges [21] concluded that radiographic markers could be a reservoir for a range of bacteria, particularly if not cleaned regularly or adequately. Similarly, Lawson et al. [25] reported a high potential for infectious organisms to thrive on diagnostic imaging cassettes, with no noticeable reduction in growth throughout entire test period (2 weeks). Ohara et al. [27] also demonstrated the capability of *Staphylococcus* species to attach to ultrasound instruments, and Ravine et al. [29] evaluated the attachment potential of bacteria on thermoplastic masks used in radiation therapy. These studies concluded that both ultrasound instruments and thermoplastic masks could harbor infectious organisms, however the tested bacteria did not appear to survive for long [27,29]. All four studies highlight the ability for infectious microorganisms to attach and survive on MRS equipment, indicating a need for appropriate infection control methods to prevent the occurrence of OAIs. 

### 4.4. Limitations

While recognizing that SRs allow for a more objective appraisal of available evidence in comparison to integrative, narrative, or scoping reviews, they can have a number of limitations. First, they cannot pick up all the articles that may be published in any area [36,37]. In addition, they can also be subject to a number of biases [38]. According to the hierarchy of evidence, observational studies are ranked relatively low (in comparison to the “gold standard” randomized controlled trials) [33]. All 19 studies included in this review demonstrate the utilization of research designs that typically lack adequate methodological rigor to minimize the effects of bias [33]. However, the use of such a rigid hierarchy is being questioned by some authors such as Concato [39], who argue that evidence-based hierarchies overstate the limitations of observational studies [39]. The majority of studies evaluating the effectiveness of such a hierarchy have concluded that the importance of a study design depends on the research question being studied [33,39]. A rigid hierarchy of evidence may not be well suited for microbiological research, where the aim is observing the levels of organisms that are living within an environment [40]. Randomized controlled trials are often not an appropriate study design for this type of research as an intervention is not applied [39,40]. As such, applying a rigid design hierarchy to research of this nature is potentially less important than evaluating the rigor of the study’s methodology [40].

Of the 19 studies included in this SR, 10 used samples involving less than 50 subjects. Additionally, only five of those studies provided justification for their sample size. Smaller sample sizes increase the probability of a sample returning a non-significant result [39], which makes it more difficult to determine the true levels of infectious organisms within MRS departments and limits the application of results to the wider population. 

Several included studies failed to randomly select subjects, further limiting the ability for findings to be extrapolated to a wider population. Six of the 19 studies used random selection, three used convenience sampling, and the remaining 10 did not state their sampling procedure. Without sufficient sampling information, it is difficult to determine whether sampled equipment is unbiased and representative of the equipment most commonly used in MRS departments. Several studies also incorporated a questionnaire into their research or recruited samples over several months, and some studies failed to report whether participating professionals were blinded to the nature of the study [15,22]. Without being blinded, subjects may have been inclined to clean their personal equipment (radiographic markers, scissors, etc.) more regularly, thus directly affecting the reported level of contamination. 

The ability to extrapolate results even to other MRS departments (let alone broader healthcare) is difficult as only two studies were set in radiation therapy departments, and there are no studies set in nuclear medicine departments. Whilst the results were relatively consistent across the settings of included studies, further research is necessary to determine whether the findings reported in this review can be applied to other MRS departments. 

Variable methods of data collection were used across the included studies, including different swabbing techniques and culturing mediums. Levels of contamination were also reported differently, with one study reporting the results solely in terms of CFUs rather than actual infectious organisms [6]. There were also discrepancies between which organism(s) each study was testing for, as well as variations in classifications of reported organisms. Due to these factors, it is possible that the true prevalence of infectious organisms in MRS may be underestimated. 

Most studies classify coagulase-negative *Staphylococcus* as a significant pathogen, despite it being part of the environmental skin flora [28]. However, certain studies, such as Ota et al. [28], excluded environmental flora from their results to prevent overestimation of contamination. Accordingly, this study also reported equipment with the lowest levels of contamination (13.6%). With the inclusion of environmental flora, bacterial contamination was reported on 96.6% of samples [28]. This result increases the pooled mean level of contamination from 62.5% to 68%. This discrepancy in reporting across studies could result in either an underestimation or an overestimation of potentially infectious organisms. Given the nature of opportunistic pathogens and the high-risk environment of MRS, healthcare professionals should be mindful when applying infection control strategies to minimize the occurrence of OAIs. 

### 4.5. Research Gaps and Avenues for Further Research

Based on this SR, a number of research gaps have been identified.

1. No studies have been published on infectious organisms within nuclear medicine departments, thus the risks of exposure for nuclear medicine staff and the potential of nuclear medicine equipment to be vectors for OAIs remains unknown. Further research needs to be conducted to address this gap and assess the correlation to other MRS professions.
2. No studies were undertaken and published from Australia or any South American countries. Further research needs to be undertaken to determine whether the infectious organisms identified within this study or different organisms are present in Australia and South America.
3. The reservoirs of infectious microorganisms identified in the SR are the basis for experimental studies to assess the risk and burden of OAI exposure specifically in nuclear medicine departments and among MRS staff and in MRS equipment.
4. The literature has not identified if there is common OAI exposure risks when comparing MRS staff and other allied health professionals OAI risk. Further empirical research needs to be undertaken within diagnostic radiography, nuclear medicine, and radiation therapy departments and to be compared to differing allied health professions.

## 5. Conclusions

This SR aimed to identify the infectious organisms that may be found in MRS, their reservoirs, and modes of transmission. A range of different infectious organisms were identified in the literature, including Gram-positive bacteria, Gram-negative bacteria, and fungi. Equipment utilized in MRS departments, such as radiographic cassettes and ultrasound probes, was demonstrated to be a potential reservoir for bacteria and a source of OAIs. As a vast majority of the infectious organisms identified are transmissible by contact, appropriate infection control strategies are imperative to minimize the number of infectious organisms on MRS equipment and surrounding surfaces. Reducing the number of infectious organisms within MRS departments minimizes the risk of OAIs, and also decreases the risk of these infectious organisms being transferred to patients and the wider community. 

Only two of the included studies were set in radiation therapy departments and no studies were set in nuclear medicine departments, limiting the generalizability of the results. Further research would be recommended in order to provide clinically meaningful results that can be extrapolated to all MRS professions. 

## Figures and Tables

**Table 1:** The search strategy performed in one of the five databases (Medical Literature Analysis and Retrieval System Online; MEDLINE).

#	Searches
1	healthcare associated infection/
2	(“hospital acquired infection *” or “healthcare associated infection*” or hospital pathogens or “nosocomial”).mp. [mp = title, abstract, original title, name of substance word, subject heading word, floating sub-heading word, keyword heading word, organism supplementary concept word, protocol supplementary concept word, rare disease supplementary concept word, unique identifier, synonyms]
3	1 or 2
4	exp Allied Health Occupations/ or exp Allied Health Personnel/
5	(“radiation therap *” or “radiotherap*” or “allied health” or “nuclear medicine” or “molecular imag*”).mp. or radiography/ or “medical imaging”.mp. [mp = title, abstract, original title, name of substance word, subject heading word, floating sub-heading word, keyword heading word, organism supplementary concept word, protocol supplementary concept word, rare disease supplementary concept word, unique identifier, synonyms]
6	4 or 5
7	3 and 6
8	limit 7 to English language
9	Limit 8 to year = ”1983–2018”

**Table 2:** Characteristics of included studies.

Reference	Design	Sample	Setting	Equipment Examined	Infectious Organisms Identified (*n*)
Akpochafor et al. (2015) [14]	CO	36	DR (US)	Ultrasound probes, couches, coupling gel	P = 3 N = 3 F = 2
Arora et al. (2009) [15]	CS	160	NS	Mobile phones	P = 6 N = 4
Bhat et al. (2011) [16]	CS	204	NS	Mobile phones	P = 5 N = 7
Boyle and Strudwick (2010) [17]	CS	15	DR	Lead rubber aprons	P = 4
Brewer et al. (2014) [18]	CS	24	RT	Heating appliances	P = 3
Denis et al. (2003) [19]	CO	814	DR	n/a	n/a
Fox and Harvey (2008) [20]	CS	40	DR	Radiographic cassettes	P = 4
Giacometti et al. (2004) [6]	CS	144	DR	X-ray tubes, control panels, radiographic cassettes, imaging plates	n/a
Hodges (2001) [21]	CO	10	DR	Radiographic markers	P = 6 N = 4 NS = 1
Kelly and Trundle (2014) [22]	CS	100	Nursing	Pocket scissors	P = 5 N = 1
Kiran et al. (2018) [23]	CO	98	DR (US)	Ultrasound probes, coupling gel	P = 3
LaBan et al. (2004) [24]	CS	14	RU	Patient charts	P = 1
Lawson et al. (2002) [25]	QE	3	DR	Imaging cassettes	P = 2 N = 1
Ochie and Ohagwu et al. (2009) [26]	CO	301	DR	X-ray couches, chest stands, radiographic cassettes, handles of x-ray tube heads, control panels, exposure buttons, patient x-ray gowns	P = 2 N = 2
Ohara et al. (1998) [27]	QE	9	DR (US)	Ultrasound probes	P = 2 N = 2
Ota et al. (2007) [28]	CS	118	NS	Identification badges	P = 6 N = 5 F = 1
Ravine et al. (2017) [29]	QE	4	RT	Thermoplastic immobilization masks	P = 2 N = 2
Ridge (2005) [30]	CS	28	DR (US)	Ultrasound probes, gel bottle tips, pressure cuffs	P = 4 N = 1 F = 1
Tugwell and Maddison (2011) [31]	CS	50	DR	Radiographic markers	P = 4

CO, Cohort; CS, Cross-Sectional; DR, Diagnostic Radiography; F, Fungi; N, Gram-Negative Bacteria; NS, Non-Specific; P, Gram-Positive Bacteria; QE, Quasi-Experimental; RT, Radiation Therapy; RU, Rehabilitation Unit; US, Ultrasound.

**Table 3:** Genera of healthcare-associated infectious organisms identified in the literature and their common route/s of transmission.

Gram-Positive (*n* = 7)	T	Gram-Negative (*n* = 10)	T	Fungi (*n* = 2)	T
*Staphylococcus*	C	*Acinetobacter*	C	*Candida*	B/C
*Micrococcus*	C	*Pseudomonas*	C	*Cladosporium*	C
*Kocuria*	B	*Escherichia*	C		
*Bacillus*	A/C	*Klebsiella*	C		
*Streptococcus*	A/C	*Enterobacter*	C		
*Enterococcus*	C	*Citrobacter*	C		
*Corynebacterium*	A/C	*Proteus*	C		
		*Neisseria*	A/C		
		*Moraxella*	C		
		*Vibrio*	C		

A, Airborne; B, Bloodborne; C, Contact; T, Mode of Transmission.

**Table 4:** Pooled range and mean for each outcome measure investigated [32].

Outcome Measure	Pooled Range	Pooled Mean
Percentage of contamination of equipment	13.6–100	62.5
Number of colony-forming units present on sampled equipment	0–1000	82.6
Number of different genera on sampled equipment	1–10	5

**Table 5:** Methodological quality of included studies.

Study	Critical Appraisal Checklist Item Number											Outcome
	1	2	3	4	5	6	7	8	9	10	11	
Akpochafor et al. (2015) [14]	Y	Y	Y	Y	Y	Y	Y	Y	Y	Y	Y	Y
Arora et al. (2009) [15]	Y	Y	Y	Y	U	U	Y	Y	-	-	-	Y
Bhat et al. (2011) [16]	Y	Y	Y	Y	Y	Y	Y	Y	-	-	-	Y
Boyle and Strudwick (2010) [17]	Y	Y	N	Y	Y	Y	U	U	-	-	-	Y
Brewer et al. (2014) [18]	Y	Y	Y	Y	Y	Y	U	Y	-	-	-	Y
Denis et al. (2003) [19]	Y	Y	Y	Y	Y	Y	Y	Y	Y	Y	Y	Y
Fox and Harvey (2008) [20]	Y	Y	Y	Y	Y	Y	U	Y	-	-	-	Y
Giacometti et al. (2004) [6]	Y	Y	Y	Y	Y	Y	Y	Y	-	-	-	Y
Hodges (2001) [21]	Y	Y	Y	Y	Y	Y	Y	Y	U	Y	Y	Y
Kelly and Trundle (2014) [22]	Y	Y	Y	Y	Y	Y	Y	Y	-	-	-	Y
Kiran et al. (2018) [23]	Y	Y	Y	Y	Y	Y	Y	Y	Y	Y	Y	Y
LaBan et al. (2004) [24]	Y	Y	U	Y	U	U	U	Y	-	-	-	U
Lawson et al. (2002) [25]	Y	Y	Y	Y	Y	Y	Y	U	Y	-	-	Y
Ochie and Ohagwu et al. (2009) [26]	Y	Y	Y	Y	U	Y	Y	Y	Y	Y	Y	Y
Ohara et al. (1998) [27]	Y	Y	Y	Y	Y	Y	Y	U	Y	-	-	Y
Ota et al. (2007) [28]	Y	Y	Y	Y	Y	Y	Y	Y	-	-	-	Y
Ravine et al. (2017) [29]	Y	Y	Y	Y	Y	Y	Y	U	Y	-	-	Y
Ridge (2005) [30]	Y	Y	Y	Y	Y	Y	Y	Y	-	-	-	Y
Tugwell and Maddison (2011) [31]	Y	Y	Y	Y	Y	Y	U	Y	-	-	-	Y

* All studies appraised with the JBI checklist appropriate to the study design [13]. N, No; U, Unclear; Y, Yes.
